# Amino-acid PET versus MRI guided re-irradiation in patients with recurrent glioblastoma multiforme (GLIAA) – protocol of a randomized phase II trial (NOA 10/ARO 2013-1)

**DOI:** 10.1186/s12885-016-2806-z

**Published:** 2016-10-05

**Authors:** Oliver Oehlke, Michael Mix, Erika Graf, Tanja Schimek-Jasch, Ursula Nestle, Irina Götz, Sabine Schneider-Fuchs, Astrid Weyerbrock, Irina Mader, Brigitta G. Baumert, Susan C. Short, Philipp T. Meyer, Wolfgang A. Weber, Anca-Ligia Grosu

**Affiliations:** 1 Department of Radiation Oncology, Medical Center – University of Freiburg, Faculty of Medicine, Robert-Koch-Str. 3, 79106 Freiburg, Germany; 2 Department of Nuclear Medicine, Medical Center – University of Freiburg, Faculty of Medicine, Hugstetterstraße 55, 79106 Freiburg, Germany; 3 Clinical Trials Unit, Medical Center – University of Freiburg, Faculty of Medicine, Elsässer Straße 2, 79110 Freiburg, Germany; 4 German Cancer Research Center (DKFZ), Heidelberg, Germany; 5 German Cancer Consortium (DKTK), Partner Site Freiburg, Germany; 6 Department of Radiation Oncology, St. Josef’s Hospital, Weingartenstraße 70, 77654 Offenburg, Germany; 7 Department of Neurosurgery, Medical Center – University of Freiburg, Faculty of Medicine, Breisacher Str. 64, 79106 Freiburg, Germany; 8 Department of Neurosurgery, Cantonal Hospital St. Gallen, Rorschacher Str. 95, CH-9007 St. Gallen, Switzerland; 9 Department of Neuroradiology, Medical Center – University of Freiburg, Faculty of Medicine, Breisacher Straße 64, 79106 Freiburg, Germany; 10 Department of Radiation Oncology, MediClin Robert Janker Clinic & Cooperation Unit Neurooncology, University of Bonn Medical Center, Villenstr. 8, 53129 Bonn, Germany; 11 Department of Radiation Oncology (MAASTRO) & GROW (School for Oncology & Developmental Biology), Maastricht University Medical Center, Maastricht, The Netherlands; 12 Clinical Oncology, Leeds Cancer Centre, St James’s University Hospital, Leeds Teaching Hospitals NHS Trust, Leeds, UK; 13 Department of Radiology, Molecular Imaging and Therapy Service, Memorial Sloan Kettering Cancer Center, 1275 York Avenue, New York, NY 10065 USA; 14 Leeds Institute of Cancer and Pathology, University of Leeds, St James’s University Hospital, Leeds, UK

**Keywords:** Amino-acid PET, T1-Gd-MRI, Re-irradiation, Recurrent glioblastoma

## Abstract

**Background:**

The higher specificity of amino-acid positron emission tomography (AA-PET) in the diagnosis of gliomas, as well as in the differentiation between recurrence and treatment-related alterations, in comparison to contrast enhancement in T1-weighted MRI was demonstrated in many studies and is the rationale for their implementation into radiation oncology treatment planning. Several clinical trials have demonstrated the significant differences between AA-PET and standard MRI concerning the definition of the gross tumor volume (GTV). A small single-center non-randomized prospective study in patients with recurrent high grade gliomas treated with stereotactic fractionated radiotherapy (SFRT) showed a significant improvement in survival when AA-PET was integrated in target volume delineation, in comparison to patients treated based on CT/MRI alone.

**Methods:**

This protocol describes a prospective, open label, randomized, multi-center phase II trial designed to test if radiotherapy target volume delineation based on FET-PET leads to improvement in progression free survival (PFS) in patients with recurrent glioblastoma (GBM) treated with re-irradiation, compared to target volume delineation based on T1Gd-MRI. The target sample size is 200 randomized patients with a 1:1 allocation ratio to both arms. The primary endpoint (PFS) is determined by serial MRI scans, supplemented by AA-PET-scans and/or biopsy/surgery if suspicious of progression. Secondary endpoints include overall survival (OS), locally controlled survival (time to local progression or death), volumetric assessment of GTV delineated by either method, topography of progression in relation to MRI- or PET-derived target volumes, rate of long term survivors (>1 year), localization of necrosis after re-irradiation, quality of life (QoL) assessed by the EORTC QLQ-C15 PAL questionnaire, evaluation of safety of FET-application in AA-PET imaging and toxicity of re-irradiation.

**Discussion:**

This is a protocol of a randomized phase II trial designed to test a new strategy of radiotherapy target volume delineation for improving the outcome of patients with recurrent GBM. Moreover, the trial will help to develop a standardized methodology for the integration of AA-PET and other imaging biomarkers in radiation treatment planning.

**Trial registration:**

The GLIAA trial is registered with ClinicalTrials.gov (NCT01252459, registration date 02.12.2010), German Clinical Trials Registry (DRKS00000634, registration date 10.10.2014), and European Clinical Trials Database (EudraCT-No. 2012-001121-27, registration date 27.02.2012).

## Background

During the last years enormous progress has been made in the area of high precision radiotherapy [1]. In the brain it is now technically feasible to irradiate complex target volumes with a precision of less than 1 mm, while sparing normal tissues [2]. This offers the opportunity to significantly escalate the radiation dose for the tumor tissue, which is considered to be a key for increasing local control rates. However, the potential of high precision radiotherapy can only be realized when the tumor volume can be accurately delineated by imaging techniques [3]. Studies have shown that standard anatomic imaging modalities (CT, MRI), while very accurate at visualizing normal anatomical structures, are limited in defining tumor extension for radiation treatment planning [4]. Traditionally, the target volume definition for irradiation, as well as re-irradiation after recurrence, of malignant gliomas is based on T1-weighted MRI with Gadolinium (Gd) [5]. Contrast enhancement is a consequence of disruption of the blood-brain barrier (BBB) which does not necessarily reflect the real tumor extension in gliomas. Gross tumor mass has been detected beyond the margins of contrast enhancement, in the surrounding edema and even in the adjacent normal-appearing brain tissue [6–10].

After therapy (surgery, irradiation and/or chemotherapy), BBB disturbances can frequently be treatment-related (for example associated with postoperative granulation or radiation necrosis) and cannot be differentiated from persistent tumor on conventional MRI [9]. This phenomenom was termed “pseudoprogression” [11] and, in this case non-tumoral tissue may be erroneously included in the gross tumor volume (GTV), leading to a higher rate of sides effects after re-irradiation. Vice versa, after systemic treatment with vascular endothelial growth factor (VEGF) receptor signalling pathway inhibitors such as bevacizumab, “pseudoresponse” has been described [11–13].

In patients that have been previously irradiated, the volume of normal tissue included in high dose areas should be as small as possible [14] to avoid severe toxicities, such as radiation necrosis [15]. Therefore, the target volume has to encompass mainly the macroscopic tumor mass (GTV) without including a large area of suspected microscopic tumor infiltration (clinical target volume, CTV). The margins of the planning target volume (PTV) have to be very small, in order to spare normal brain tissue. High conformal radiation strategies like stereotactic-fractionated radiotherapy (SFRT), image-guided radiotherapy (IGRT) and intensity modulated radiotherapy (IMRT) are used to focus the irradiation on the gross tumor mass and reduce the required margin, making GTV delineation in this case a major issue [9, 16, 17].

For high precision radiotherapy, inaccuracies in tumor delineation may offset any gain in local control rates achieved by dose escalation, emphasizing the need for new imaging approaches to increase tumor delineation for high precision radiotherapy [18].

Along this line, imaging the biological and molecular characteristics of the tumor tissue by positron emission tomography (PET) is an interesting approach to improve treatment planning for high precision radiotherapy. Multiple studies correlating imaging findings with histopathological evaluation in surgically treated patients with high grade glioma have indicated that molecular imaging with amino acid (AA) PET (*L*-[methyl-^11^C]methionine (MET) or *O*-(2-[^18^F]fluoroethyl)-L-tyrosine (FET)) is more specific and equally sensitive for tumor staging than MRI [4, 19]. Based on these data, the infrastructure for AA-PET imaging has become widely available in major hospitals [20]. Although AA-PET imaging shows great promise for target delineation, it has not been rigorously evaluated in clinical trials. Several studies in patients with gliomas have indicated that PET based target volumes differ markedly from target volumes defined by MRI, but the methodology for tumor delineation on PET images differs significantly among these studies [8, 21–28]. More importantly, there are no randomized trials that have evaluated the impact of PET based radiotherapy on patient outcome. According to relevant clinical trial registers, no clinical trials are currently running or planned for this indication (http://www.drks.de, http://www.controlled-trials.com, http://clinicaltrials.gov, http://www.who.int/ictrp; last accessed on 07.05.2016). Generally, imaging techniques have so far not been evaluated with the same rigor as therapeutic agents. The high costs associated with modern imaging techniques make it necessary to use a similar approach as for evaluation of new therapeutic agents.

The hypothesis of the study is that AA-PET, having a higher specificity and equal sensitivity for tumor tissue in comparison to MRI (T1 with gadolinium), will visualize the tumor mass with a higher precision and thus will improve patient outcome. This hypothesis was pre-tested in a previous small prospective monocentric non-randomized pilot study led by the principal investigators [9]. In this cohort of 44 patients, a statistically significant better survival time was reported in patients with recurrent gliomas treated with re-irradiation using SFRT based on AA-PET in comparison to the same irradiation regime based on T1Gd-MRI on univariate analysis. The goal of this trial is to verify the improved outcome for patients in a randomized multicenter phase II study with progression free survival (PFS) as the primary endpoint to specifically address the potential impact of the differences in radiation target volumes. The results of this trial could have a significant impact not only on GTV delineation in recurrent GBM, but also in the tumor mass delineation of primary tumors, in the evaluation of tumor response and treatment monitoring and in developing of a standardized methodology for target volume delineation based on PET. A further goal of the study is to establish a framework for the use of molecular imaging in radiation oncology.

## Methods/design

### Trial design and setting

This is a prospective, open label randomized (allocation 1:1) two-arm parallel group phase II multi-centre trial designed to test for differences in the impact of an FET-PET-based (experimental, Arm A) versus a T1Gd-MRI-based (control, Arm B) treatment planning on the progression-free survival in patients with recurrent GBM treated with re-irradiation.

Trial sites are academic hospitals and community clinics located in Aachen, Bonn, Dessau, Erlangen, Freiburg, Hannover (two sites), Karlsruhe, Köln, Magdeburg, Mannheim, Marburg, München, Offenburg, Rostock, Stuttgart, Trier, and Tübingen.

The trial was approved by the ethics committee of the University of Freiburg (EK-Freiburg 133/10) and by the local ethics committees of participating sites. The GLIAA trial has been thoroughly examined and approved by the Federal Office for Radiation Protection (Bundesamt für Strahlenschutz, BfS) and the Federal Institute for Drugs and Medical Devices (Bundesamt für Arzneimittel und Medizinprodukte, BfArM). Written informed consent for study participation is obtained from all patients before the initiation of any study-specific procedures. The GLIAA trial is associated with the German Neurooncological Network (Neuroonkologische Arbeitsgemeinschaft, NOA-10) and Working Group Radiation Oncology (Arbeitsgemeinschaft Radiologische Onkologie, ARO2013-1) of the German Cancer Society (Deutsche Krebsgesellschaft, DKG).

### Study population

The target population for this trial is previously irradiated patients with recurrent GBM. For the proposed trial, there is no gender requirement. No gender ratio has been stipulated in this study as the results of the preclinical and/or clinical studies did not indicate any difference in the effect of the study treatment in terms of efficacy and safety. No healthy persons will be included. To avoid selection bias, investigators should enroll patients irrespectively of whether or not any differences are seen in GTV-delineation based on FET-PET versus T1Gd-MRI.

Key inclusion criteria at the time of randomization are: (1) Patient’s written informed consent (IC) obtained latest the day after FET-PET acquisition, (2) legal capacity: Patient is able to understand the nature, significance and consequences of the study, (3) age ≥ 18 years (no upper limit of age), (4) Karnofsky Performance Score (KPS) > 60 %, (5) registration in the electronic case report form, (6) recurrence of GBM (WHO grade IV) and either not eligible for tumor resection or with macroscopic residual tumor after resection of the recurrent GBM, (7) histological confirmation of GBM at initial or secondary diagnosis, (8) previous radiation therapy of high grade glioma (WHO Grad III or IV) with a total dose of 59.4 - 60Gy (single dose 1.8 – 2.0 Gy), (9) at least 6 months between the end of pre-irradiation and randomization, (10) recurrent tumor visible on FET-PET and T1Gd-MRI with the maximum diameter measuring 1 cm to 6 cm by either technique (in case of multifocal tumor, the sum of all diameters has to be 1-6 cm on FET-PET and T1Gd-MRI), (11) target volume definition possible according to both study arms, (12) start of re-irradiation planned within 2 weeks from FET-PET and MRI. Key Exclusion criteria are: (1) Recent (≤ 4 weeks before IC) histological result showing no tumor recurrence, (2) previous treatment of GBM with bevacizumab or other molecular targeted therapies less than 6 months before MRI and FET-PET used for radiotherapy planning, (3) technical impossibility to use MRI or FET-PET dataset for RT planning, (4) less than 2 weeks between the last day of last chemotherapy given and planned start of reirradiation, (5) less than 3 weeks between resection of recurrent GBM and planned start of re-irradiation, (6) chemotherapy or molecular targeted therapies planned during re-irradiation, (7) additional chemotherapy or molecular targeted therapy or further surgery planned before diagnosis of further tumor progression after study intervention, (8) simultaneous participation in other interventional trials which could interfere with this trial and/or participation in a clinical trial within the last thirty days before the start of this study and/or previous participation (randomization) in this study, (9) pregnancy, nursing, or patient not willing to prevent a pregnancy during treatment, (10) known or persistent abuse of medication, drugs or alcohol, (11) known allergy against the MRI contrast agent Gadolinium or the PET tracer ^18^F-FET or against any of the components.

### Study treatment and procedures

The trial randomizes the patients to the following two treatment arms with a 1:1 allocation ratio: In the control intervention, the MRI based GTV delineation will be performed with respect only to the T1Gd-MRI image dataset. Here, the GTV is defined as the tumor related contrast enhancement with no safety margin in accordance with a neuroradiologist. In the experimental arm, the PET based GTV delineation will employ FET uptake with a greater value than 1.8 +/- 0.1 times of normal brain tissue uptake as a starting point, visually verified and modified by an experienced nuclear medicine specialist together with the treating radiation oncologist. To define the acquisition protocol and reconstruction parameters for each participating study centre, PET/CT scanner phantom studies will be performed. Freiburg will be the reference centre.

Starting from the GTV, for both target volumes (MRI and FET-PET based), a CTV will be defined by adding 3 mm in either direction respecting anatomical boundaries like skull and/or tentorium. This CTV will then be expanded to the PTV by adding 1–2 mm in all directions.

Radiotherapy planning will be performed after randomization using the predefined target volume for the treatment arm allocated. In both study arms, patients will be given a high-precision re-irradiation with a total dose 39 Gy, 3 Gy/d, 5×/week to the PTV as defined according to the respective study arm. The dose specification will be according to the criteria of the International Commission on Radiation Units and Measurements (ICRU) with the 95 % isodose surrounding the PTV.

In both treatment arms, the MRI/CT based contouring of risk organs will be done after the definition of the GTV. The following constraints for normal tissue/organs at risk have to be respected in radiotherapy planning, which relate to the cumulative doses from the actual and the prior radiation treatment in the same irradiated point / area, calculated by the equivalent dose in 2 Gy fractions (EQD_2_, α/β = 2 Gy): (1) The maximum total dose to the optical chiasm and/or both optical nerves must not exceed 54 Gy, (2) if only one optical nerve is involved, the maximum dose must not exceed 60 Gy, (3) the maximum dose to the brain stem must not exceed 66 Gy in 10 % of volume, (4) the medulla oblongata must not receive more than 60 Gy at maximum point, (5) the maximum dose to the retina must not exceed 54 Gy.

Irradiation is given either as SFRT using an external coordinate system and/or IGRT with CT and/or kV imaging of the treatment position before every treatment fraction. The mean positioning tolerance of all fractions as documented by this imaging must be ≤ 2 mm.

**Fig. 1:**
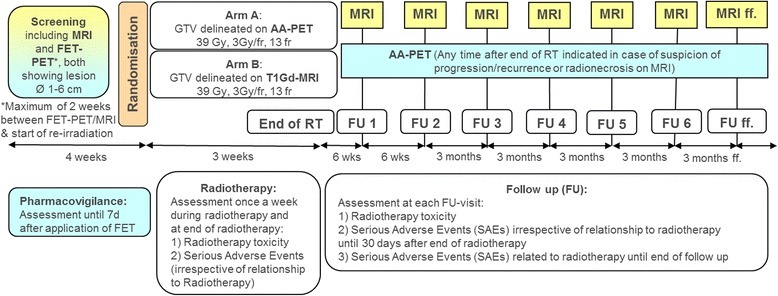
Flowchart of the GLIAA trial. AA-PET = amino acid positron emission tomography; MRI = magnetic resonance imaging; FET = *O*-(2-[^18^F]fluoroethyl)-L-tyrosine; Gd = gadolinium

The flowchart of the treatment and follow-up schedule is shown in Fig. 1.

### Quality assurance for radiation treatment planning

After completion of radiotherapy planning for the first study patient, the study centers will upload the pseudonymized PET, CT and MRI images well as the pseudonymized radiotherapy treatment plan on a dedicated web-platform. A review committee including a radiation oncologist, a nuclear medicine physician and a physicist in the coordinating study centre in Freiburg will check the key parameters of imaging and treatment planning before the initiation of radiotherapy of the second study patient. After passing quality assurance for the at least one patient, further imaging and RT planning will undergo mutual monitoring by the members of the study group in the framework of regular study group meetings.

### Study endpoints

The primary endpoint is PFS, defined as time from randomization until tumor progression or death. Progression is determined based on MRI and confirmed by AA-PET and/or positive biopsy/surgery as follows. A tumor-suspicious lesion on MRI according to RANO criteria [29] which is confirmed by AA-PET and/or biopsy/surgery is considered as tumor progression, while a negative PET-scan will exclude progression. A positive PET scan, if unclear, should be followed by biopsy. Patients receiving any new treatment (chemotherapy or immunotherapy) for progression of their GBM in the absence of diagnosed tumor progression, and patients receiving surgery for distant progression or receiving bevacizumab for a radionecrosis will also be considered as having an event for the endpoint PFS, at the date that treatment was initiated.

Secondary endpoints are the following. (1) Overall survival (OS) is defined as time from randomization to death. (2) Volumetric assessment of GTV based on PET and MRI is based on PET/MRI + RT-structure set image co-registration. The relation of both GTVs to each other, especially overlap and non-overlap volumes, will be assessed. (3) The topography of progression after re-irradiation will be evaluated at the time of progression. In all available image datasets, the topographical relation of the tumor re-growth will be scored as local progression or distant progression: Local progression will be determined as in field progression (largest proportion within the PTV), or margin progression (largest proportion within 2 cm and located in the same anatomical region as the PTV); Distant progression will be determined as outfield progression (largest proportion clearly [>2 cm] outside of PTV or located in another anatomical region). Progression will be judged as either in PET/MRI-GTV, marginal to PET/MRI-GTV, or clearly outside PET/MRI-GTV, both for PET and MRI, respectively. (4) Locally controlled survival is defined as time from randomization to local progression (see 3) or death. (5) Long term survival is defined as OS > 1 year. (6) The topography of necrosis after re-irradiation will be determined with respect to the irradiated volume (PTV) as described above. (6) Quality of life (QoL) will be determined using the EORTC QLQ-C15 PAL questionnaire [30] which is especially aimed to patients in a (near) palliative care setting. The primary scale for the QoL evaluation is the global health status/QoL scale status. The other scales and single items are secondary outcomes. The primary outcome measure is the change from baseline before radiation therapy to follow up measurements. (7) A possible impact of diffusion/perfusion and FLAIR MRI on target volume delineation will be analyzed optionally in departments having the technology and the know-how for these investigations. These evaluations will be handled as a separate subproject, to be described elsewhere. (8) Adverse events occurring until incl. day 7 after FET-PET imaging are registered to address safety of FET-PET imaging. (9) Occurrence of a range of pre-specified adverse events (AEs) considered as possible radiotherapy treatment toxicity is registered and documented according to NCI CTCAE v4.03 (http://evs.nci.nih.gov/ftp1/CTCAE/CTCAE_4.03_2010-06-14_QuickReference_5x7.pdf) during treatment and follow up. Events occurring more than 90 days after the start of irradiation are considered as late toxicity. In addition, we register serious adverse events (SAEs) occurring from start of radiotherapy until 30 days after end of radiotherapy as well as SAEs occurring during the complete follow up period which are considered as related to radiotherapy.

### Sample size

The number of patients to recruit to this phase II study is derived from the primary endpoint PFS. Based on data from a literature report [31], where 6-months PFS ranged between 28 and 39 %, and considering that this study includes also patients with larger tumors up to a diameter of 6 cm, we expect 30 % PFS at 6 months in the control arm. The objective is to detect a difference if the experimental treatment entails an increase of at least 15 % in the 6-months PFS rate. Such an improvement seems feasible, since OS in the pilot study was 5 months in the control arm (treated based on MRI) versus 9.5 months in the experimental arm (treated based on PET/SPECT) [9]. Assuming exponentially distributed PFS times, the target difference of a 15 % increase to 45 % 6-months PFS corresponds to a hazard ratio (HR) of 0.667 for the experimental versus the control group (median ratio of 1.5).

The study was planned under these assumptions, using the comparative phase II design with one-sided type I error rate α = 10 and 90 % power proposed by Korn et al. [32], and a group sequential plan with one interim analysis during the recruitment phase, with the option to stop the trial early for futility. Assuming a constant recruitment rate, the following procedure to test the null hypothesis H_0_: HR ≥ 1.0 against the alternative hypothesis of superiority in the experimental arm, H_1_: HR < 1.0, has the desired properties (SAS Version 9.2, proc sequ design): After recruitment of 115 patients over a period of 15 months, the trial is to be stopped for futility (accept H_0_) if the p-value of the one-sided log-rank test of H_0_ versus H_1_ is above 0.51742. Otherwise, recruitment is to continue for another 9 months up to a total of 184 patients over a total period of 24 months, with additional follow up for 12 months. At the final analysis after 36 months, a log-rank test of H_0_ versus H_1_ at one-sided nominal level of α = 10 % is performed. With this sequential plan, the entire procedure, accounting for the interim analysis, has a power of 90 % and a type I error rate of 9.252 %. If H_1_ is true, the probability to stop for futility is 3.021 %. Under H_1_, the expected number of events is 71.85 at 15 months and 176.22 at 36 months. To compensate for possible losses to follow up or ineligible patients, a target number of 200 randomizations over a period of 24 months was planned, and the interim analysis should be performed when 125 = 200 × 15/24) patients would be randomized.

### Randomization

Central randomization is performed by means of the minimization technique with a random element as initially described by Pocock and Simon [33], using a computerized randomizer tool (https://www.randomizer.at/) with the following factors in the minimization algorithm: (1) time since first radiation treatment, calculated between the last day of previous irradiation and randomization (≤14 months vs. >14 months) (2) previous chemotherapy treatment (≤ 7 cycles of TMZ vs. > 7 cycles) (3) maximum tumor diameter on MRI (GTV ≤ 3 cm vs. GTV > 3 cm) (4) MGMT-status (methylated vs. non-methylated vs. not yet determined). An open label design was chosen because effective blinding was considered unfeasible. However, the randomization procedure guarantees concealment of treatment allocation und thus minimizes selection bias.

### Statistical analysis

Because this is a phase II study, the primary analyses of the efficacy endpoints PFS, locally controlled survival and OS will be performed in the per protocol population, which comprises all eligible patients who started their allocated treatment (at least one radiotherapy fraction as randomized). Sensitivity analyses according to intention-to-treat will include all patients as randomized. Patients free from progression and alive at the last visit will be censored for PFS at the day of last assessment. Patients free from in-field and margin progression and alive at the last visit will be censored for locally controlled survival at the day of last assessment. Patients not known to have died during the study will be censored for OS at the day they were last known to be alive. The primary comparison of the time-to-event distributions between the two treatment arms will be done using the one-sided log-rank test at significance level α = 10 % stratified by all factors used for randomization, to test H_0_: HR ≥ 1.0 versus H_1_: HR < 1.0. The HR for the experimental versus the control arm and its two-sided 80 and 95 % confidence intervals will be estimated using a Cox proportional hazards model stratified by the same factors. Distributions of PFS, locally controlled survival and OS will be estimated by the Kaplan-Meier method. The PFS and locally controlled survival rates at six months, the OS rates at one year, and medians of PFS, locally controlled survival and OS will be presented with two-sided 95 % confidence interval computed using the log-log transformation [34]. Additional exploratory analyses will study the prognostic impact of factors other than treatment, including age, sex and MGMT status.

Exploratory QoL analyses in the per protocol population will evaluate the evolution of QoL over time in the group of patients still alive at the respective time points. The results will be interpreted in conjunction with Kaplan-Meier estimates for OS. Cross-sectional descriptions of the average scores, which range from 0 to 100, will be presented by treatment arm with confidence intervals. Missing values will be imputed via linear regression models to assess the stability of the results. The primary analyses will classify the change scores according to the established minimal clinically important difference of 10 points [35] as (a) worsening vs unimportant worsening or improvement and (b) improvement vs unimportant improvement or worsening.

Analyses of the safety of FET application are performed in the pharmacovigilance population of all patients who received FET. Analyses of radiotherapy treatment toxicity are performed in the safety population of all patients who started treatment (at least one radiotherapy fraction), irrespective of eligibility, according to the treatment arm that they started. Rates of AEs occurring until incl. day 7 after FET-PET imaging and of SAEs occurring from start of radiotherapy until 30 days after end of radiotherapy will be presented with exact two-sided 95 % confidence intervals. Further analyses of treatment toxicity will present the worst grade of acute/subacute and late side effect by treatment arm. The time to occurrence of any severe (NCI-CTC grade ≥3) side effects and to any severe late side effects will be estimated by cumulative incidence. The time to severe (late) side effects will be calculated from the time of start of radiation treatment to the first evidence of any grade 3 (late) side effects. Patients alive without grade 3 (late) toxicity will be censored at the date of last follow-up, patients who died without experiencing (late) grade 3 side effects will be assessed as competing risk at the time of death.

### Interim analyses

Initially, a first interim analysis comparing the GTV delineated according to FET uptake with the GTV delineated according to Gd enhancement in T1-weighted MRI was planned with the aim to stop the trial early for futility if the delineation would not show a difference in target volumes in a sufficient number of patients. However, in the meantime a monocenter feasibility study (German Clinical Trials Registry DRKS00000633) was started, and the protocol of the present multicenter trial was updated. It was decided that if the feasibility study would show relevant PET/MRI-GTV non-overlap in a substantial proportion of patients (> 25 %), the first interim analysis of the present multicenter trial would be cancelled. The results of the feasibility study have turned out as expected, so that the interim analysis of GTVs in the present trial will not be performed (unpublished data).

A second interim analysis is planned with the aim to stop the trial early for futility if the experimental arm shows no favourable trend in terms of the trial primary endpoint PFS. It will be performed in the per protocol population when the first 125 patients have been randomized, which was initially expected to occur at month 15 from study start. The test will be carried out as described in the paragraph on sample size. The trial will be stopped for futility (accept H_0_) if the *p*-value is above 0.51742.

Additionally, the rate of patients registered but not randomized to the study is regularly monitored descriptively during the recruitment phase. The aim is to assess the true feasibility of the study and to allow for corrective measures if there is a selection bias towards patients with large volumes or big differences in volumes not being randomized.

## Discussion

The goal of this study is to evaluate if the delineation of the target volume based on AA-PET could have an impact on the clinical outcome in patients with recurrent gliomas treated with 3D-high conformal re-irradiation.

The selection of this disease is based on several considerations. First of all, treatment of recurrent GBM is an unsolved clinical problem and the prognosis of patients has remained poor despite intense research [36]. Thus, new treatment approaches including re-irradiation are urgently needed [37]. Second, local recurrence after surgery and radiotherapy is the cause of disease progression in almost all patients. Therefore, the impact of a local treatment approach with high precision radiotherapy on patient survival is expected to be higher than for other malignant diseases, which also or predominantly recur systemically [38]. Combined with the poor prognosis of recurrent GBM, this means that the impact of molecular imaging on patient outcome can be studied in a relatively small patient population and with a relatively short follow-up period. Third, in patients treated with re-irradiation the dose should be focused on the GTV, sparing the normal brain tissue as much as possible [14]. The treatment should be performed using high-precision radiation therapy, which is able to focus the dose on the macroscopic tumor mass and to spare the normal brain tissue [2]. The accuracy in the GTV definition should have a significant impact on local tumor control and should translate into improved clinical outcome [9]. Forth, there is considerable evidence that current approaches for target delineation in recurrent GBM are limited due to unspecific treatment related changes seen on CT and MRI [4]. Fifth, there is substantial data that PET imaging with radiolabeled amino acids provides more accurate information about tumor extension than MRI or CT, especially after pretreatment [39]. Sixth, the reported differences in tumor extension observed between MRI and AA-PET, the so-called non-overlap volume of the GTVs, are significant and appear robust enough to be tested in a multicenter clinical trial [8 and unpublished data from a monocentric feasibility study]. The magnitude of the differences between AA-PET and MRI also makes it clinically highly relevant to test AA-PET based radiation treatment planning in a clinical trial. Considering the reported high sensitivity and specificity of AA-PET for detection of recurrent GBM and the large differences in tumor extension between PET and MRI observed in patients scheduled to undergo radiotherapy, there appears to be a high risk that large parts of the tumor mass are missed when radiotherapy is based on the findings on MRI only. Finally, in preparation of this randomized multicenter trial, the principal investigators performed a small monocenter non-randomized study showing that survival is significantly improved when AA-PET is implemented into radiation treatment planning [9].

Taken together, considering that in patients treated with re-irradiation we will focus the dose on the GTV, using very small margins to CTV and PTV, we consider that this is a valid clinical model for demonstrating the differences between AA-PET and T1-Gd-MRI in the visualization of GTV, and the consequences on the clinical outcome. As outlined above, we believe that recurrent GBM represents an ideal model to study the impact of molecular imaging on patient management in a randomized clinical trial.

Furthermore, the lessons from this study could also be extrapolated to primary gliomas. The results could have a significant impact on tumor mass detection, treatment planning (surgery, radiation therapy, chemotherapy, immunotherapy, gene-virotherapy which needs to be directed towards active tumor areas, etc.). An accurate visualization of tumor mass will have a significant impact on the evaluation of tumor response after treatment and on treatment monitoring and could lead to a “modified Response Evaluation Criteria In Solid Tumors (RECIST)” or RANO definition adapted for PET imaging in neurooncology.

Therefore, we consider that the results of this study could change significantly the treatment strategy in brain tumors, if in addition confirmed in a phase III trial. Another important goal of this trial is to develop a strategy for GTV delineation based on PET. Considering the increasing impact of PET in radiation treatment planning, we consider that our trial will have a significant impact on the development of systematic strategies for tumor delineation based on PET. The lessons from this study will be generally useful for the development of a biological based treatment planning, also for other tumor entities. Additionally, in the context of the pharmacovigilance aspects of this study, the safety of FET application in clinical routine as AA-PET-tracer will be evaluated.

## Abbreviations

AA: Amino acid
CT: Computed tomography
CTV: Clinical target volume
EQD_2_: Equivalent dose in 2 Gy fractions
FET: *O*-(2-[^18^F]fluoroethyl)-L-tyrosine
FU: Follow up
GBM: Glioblastoma
Gd: Gadolinium
GTV: Gross tumor volume
Gy: Gray
HR: Hazard ratio
MET: *L*-[methyl-^11^C]methionine
MGMT: O-6-methylguanine-DNA methyltransferase
MRI: Magnetic resonance imaging
OS: Overall survival
PET: Positron emission tomography
PFS: Progression free survival
PTV: Planning target volume
QoL: Quality of life
RANO: Response Assessment in Neurooncology
RECIST: Response Evaluation Criteria In Solid Tumors
RT: Radiotherapy
SAE: Serious adverse event
SFRT: Sterotactic fractionated radiotherapy
SPECT: Single-photon emission computed tomography
TMZ: Temozolomide
